# Embodied Ethics: Phenomenology of the NICU Nurse’s Touch

**DOI:** 10.1177/10497323211005434

**Published:** 2021-09-12

**Authors:** Gillian Lemermeyer

**Affiliations:** 1 University of Alberta, Edmonton, Alberta, Canada

**Keywords:** relational ethics, touch, nurse, embodied, NICU, neonatology, newborn, qualitative, phenomenology, Canada

## Abstract

This study was a phenomenological exploration of the ethics of the nurse’s touch in the Neonatal Intensive Care Unit (NICU). I explore several examples of touching encounters as gathered from NICU nurses through interview and observation, and organize the lived meanings around several thematic statements. These include the learning touch: finding a way to hold the baby, the marking touch: when touch lingers long after physical contact, the missing touch: touching without physical contact, the gnostic touch: the possibility of knowing an other and ourselves, and the call of touch: drawn to hold. Exploring the touching gestures of NICU nurses discloses the relational ethics inherent to caring practices. By attempting to articulate these practices, the hope is that the significance and contribution of the nurse’s touch might be recognized and brought forward to our individual and professional consciousness, conversations, and curricula.

Important and difficult discussions of health ethics often revolve around weighing the moral correctness of clinical actions around issues such as decision making for medical interventions, truth-telling following medical error, and informed consent for experimental therapies. Yet, in the clinical day-to-day practice, health ethics do not unfold from abstract moral discourse but instead arise from the everyday and extraordinary moments of contact between health providers, patients, and their families. These moments might better be described as revealing the felt sense of ethics that is experienced in an encounter with a vulnerable other. Many such encounters in health care are experienced in physical contact. The touch of the nurse is essential to perform routine and urgent tasks as well as to comfort and communicate with people in care. There are few patients that need a more delicate, sensitive, and skillful touch than premature or ill babies requiring care in a Neonatal Intensive Care Unit (NICU).

Touch is deeply embedded into NICU nursing, sometimes so taken-for-granted as to seem invisible, although implicated in nearly every nursing gesture and pursuit. Inserting an intravenous line, bathing a baby, assessing vital signs, or holding and rocking a baby to sleep, and more; all of these activities are rich with the immediacy and intimacy of personal contact, and dependent on sensitive, capable touch. Understanding touch as physical contact alone may fail to capture what is encountered in the touch between nurse and child. The nurse’s touch is probably one of the most simple, recognizable, and understandable gestures of NICU treatment. Indeed, the baby may not survive without it.

A phenomenological inquiry explores an experience in context, as it is lived in the world. Michel Serres (2008) suggests that the world is not a medium in which we live, rather, that things in the world mingle with each other. This includes us, intersecting and caressing the world, as it intersects with and caresses us. Over the last months, the world within which we mingle, intersect, and caress is fundamentally changing. With the onset of a global COVID-19 pandemic, our lives and the way we live them are radically transformed. Undertaking “social” or “physical” distancing has become an unvarying public health directive and the theme of daily life. We are being educated, and legislated, to stay apart from each other. The nature of the novel coronavirus’ contagion makes our intersections with those we know and those we do not starkly clear; we breathe each other’s breath and our touches linger on surfaces and packages long after we are gone from them. Due to the risk of disease transmission, physical contact has become associated with a danger of becoming ill or transmitting disease.

Many health care practitioners, including NICU nurses, are not able to physically distance themselves from the babies that need their care. The interviews and observations in this study did not occur during the pandemic and do not address “the dangerous touch,” nor discuss the ways touching practices are currently being mediated by personal protective equipment or legislated behavior changes. Still, the current global situation allows for a unique methodological possibility as we enter into this text, writer and reader. Missing the ordinary and extraordinary experiences of touch in our lives has given rise to a heightened awareness of human touch between people and what it can mean to us all. An increased sensitivity about human touch and its meanings in our lives in general unsettles our pre-understandings and preconceived biases about the nurse’s touch in the NICU. Phenomenologically, we have become shaken from our natural attitude, released in part from the state of moving through the world without questioning or even noticing those everyday practice experiences.

## Related Literature

Human touch, as a tool or technique of nursing practice, has been written about in adult patient settings for decades. Research studies are somewhat limited and sporadic. Early on, observational studies counted and categorized nursing touch as variations of necessary (or procedural) and non-necessary (or communicative and caring) (Barnett, 1972; Mitchell et al., 1985; Watson, 1975). An assumption in many of these studies is that the touches of nurses exist in one category or another, without making clear how different types of touches are identified in research or occur in practice (Estabrooks & Morse, 1992; O’Lynn & Krautscheid, 2011). The research results tend to offer information about who touches who, where, and how often, without uncovering any real sense of the possible meanings of touch in nursing practice (Estabrooks & Morse, 1992; Jones & Yarbrough, 1985).

This may in part be due to the notion of nursing touch not being clearly defined in the literature (Connor & Howett, 2009; Estabrooks, 1987; Gleeson & Timmins, 2005; Warwick, 2017). Often a conventional definition of touch as observable skin-to-skin contact can limit studying other variations of touch (Estabrooks & Morse, 1992). Using grounded theory methods to consider the process of nursing touch, Estabrooks and Morse (1992) discuss touch as a tactile gestalt, involving other sensual components such as voice, movement and gaze.

As well as necessary to provide physical care, human touch is an important form of non-verbal communication (Chang, 2001; Gleeson & Timmins, 2004; Kübler-Ross, 2014) and can be used to demonstrate empathy (Kelly et al., 2020), help patients to cope (Bottorff, 1993), and “may well be one of the most central aspects of healing that occurs between a nurse and a patient” (Connor & Howett, 2009, p. 127). Touching encounters reveal the ethics embodied between nurse and patient and make possible a relational space where openness and disclosure are made possible (Benner, 2004). Here, touch can be a transformative experience for both patient and nurse (Edvardsson et al., 2003). In a technical and busy health care environment, the nurse’s touch is a means of re-establishing mutuality between nurse and patient that is the moral foundation of care (Gadow, 1984).

Intimate touch of patients’ bodies is a routine part of accomplishing many of the tasks of nursing (Adomat & Killingworth, 1994; Chang, 2001; Connor & Howett, 2009; Estabrooks & Morse, 1992; Pedrazza et al., 2018). Patients expect to be touched intimately and that the nurse knows what to do (Edwards, 1998). On the contrary, nurses tend to be more comfortable initiating touch than being touched (Edwards, 1998), which is similar across health disciplines (Kelly et al., 2018). It is possible that nurses feel differently related to their care settings; Routasalo (1996) discovered that it was important for nurses to receive non-necessary touches, such as hugs, from residents in elder care. Ambivalence and uncertainty about physically touching patients can be related to several factors (O’Lynn & Krautscheid, 2014; Pedrazza et al., 2015; Picco et al., 2010). For example, gender is one barrier, with evidence to suggest male nurses feel more discomfort and worry about how their touch will be interpreted by patients (Pedrazza et al., 2018; Whiteside & Butcher, 2015).

Ambivalence and uncertainty around touching may be related to gaps in nursing undergraduate education. Little is known about how nursing students learn about touch during their education (O’Lynn & Krautscheid, 2014) and nurses have acknowledged a desire for more explicit education on touch (Estabrooks & Morse, 1992). Adult patients have indicated the belief that touching should be taught in basic nurse education (Mulaik et al., 1991). Nurses identify the workplace as the place they learn to touch, through trial and error (Estabrooks & Morse, 1992; Paterson et al., 1996). Beyond simply how to touch, a lack of education addressing the nurse’s own emotional responses to touch may potentially leave them unsure and ill-equipped to respond to patients’ needs (van Dongen & Elema, 2001). While the research and commentary focused on nurse touching encounters with adult patients can offer opportunities to contemplate ethical moments and meaning, it is insufficient to simply “apply” it to the particularly vulnerable premature and ill newborn babies of the NICU.

### The Nurse’s Touch in the NICU

Human touch is widely considered critical in the development of infants and children and throughout the human life span (Barnard & Brazelton, 1990; Colton, 1983; Field, 2014; Montagu, 1986). Premature infants often lack tactile stimulation and comforting touch, beginning with their early birth (Abdallah et al., 2013; Field, 2014). Following admission to the NICU, and especially if unstable, the babies may be touched in ways that are unsupportive to their development and comfort (Álvarez et al., 2017; Benner, 2004).

Babies in the NICU undergo various more or less invasive procedures for diagnostic and therapeutic purposes, in an often brightly lit, loud, and otherwise inappropriately sensory overloaded environment (Abdallah et al., 2013; Im & Kim, 2009; Smith, 2012; Zeiner et al., 2016). Excessive noise and long-term exposure to pain can have detrimental effects on the physical and behavioral development of premature babies (Brown, 2009; Lidow, 2002). Policies of using minimal touch—limiting the touching of the baby to prevent infection and to minimize unpleasant or procedural touch may paradoxically contribute to both sensory deprivation (curtailing comforting and supportive touching) and overstimulation (procedural touching) (Álvarez et al., 2017; Leonard, 2008; Smith, 2012).

The NICU nurse’s experience of touching the baby and their families is a relatively unexplored subject, particularly the possible meanings of the ethical encounter of nurse and baby. This is despite the need for babies to be cared for through touch for even their most basic needs and the importance of touch to growth and development. This research project explores the relational ethics of health care practice by considering the phenomenology of a lived experience: the touching encounter of nurse and baby. To help realize its significance in NICU nursing care and to grasp an even deeper human meaning, I explore the lived experience of the nurse’s touch as an exemplar of embodied, originary ethics in nursing in the NICU.

## Methodology

Touch between people is a human event overflowing with complicated, contextual, contemporaneous meanings and it would be foolish and unhelpful not to acknowledge the many implications and associations that are lugged along with the word touch. This is especially true in an age where the abuses of touch are (finally) coming more fully to light and the risks of touch seem heightened by a pandemic response requiring us to be physically distant from one another. These are not the experiences of touch I seek to understand here but rather I am deliberately oriented to exploring the lived experience of touch by the NICU nurse.

I employed the qualitative research method phenomenology of practice, a context-sensitive form of interpretive inquiry (M. van Manen, 1997, 2014). It is an approach well-suited to cultivate rich and ethically sensitive understandings of human life as it is lived. The ethical meaning of touch between nurses and babies or their parents cannot be separated from the context within which it takes place. Different from theorizing or explaining touch, a phenomenological approach allows us to describe, regard, and grasp a deeper understanding of touch as it is lived through.

Phenomenology of practice is grounded in the phenomenological philosophy of Husserl, Heidegger, Merleau-Ponty and others, and the practical phenomenology of the Utrecht school (M. van Manen, 2014). The Utrecht scholars were practitioners, psychologists, physicians, and teachers who saw the possibility of an applied use of phenomenology that could lead to better, more reflective practice and practitioners. It draws together the philosophical concern of understanding life as lived with the orientation of practice, an understanding rooted in the ordinary and everyday. To understand experience, it is not enough to have it explained or summarized or analyzed. Rather, coming to know another’s experience requires a sympathy or resonance with the other that can help to foster an understanding of what their experience was like and what it might mean. Phenomenology makes possible this kind of pathic, tentative understanding, through reflection on pre-reflective experience, before we have had time to analyze and label the experience, or attach it to a theory or other preconception. Phenomenology of practice assists us to draw near to a human experience, to try to understand it better, and to “nurture a measure of thoughtfulness and tact in the practice of our professions” (M. van Manen, 2014, p. 31).

The underlying notion of this study is that the touching encounter between the nurse and patient is one of relational, embodied ethics. While acknowledging the utility of moral philosophy and rational approaches to ethics, relational ethics is founded on the premise that life is lived together with others. In their relational ethics project, Bergum and Dossetor (2005) phenomenologically explore thematic ideas such as complexity, vulnerability, uncertainty and environment. They emphasize that relational ethics is an embodied ethics; we live ethics in our relationships with others. I hope to deepen and explore this idea here. Another influence is Maurice Merleau-Ponty’s (2012) phenomenology of the body and perception. The realization that we experience the world through and as our bodies helps us understand ethics as occurring not at a distance, or in a rational way, but enmeshed in the particular, “which locates the ethical moment within the beckoning of the body” (Mazis, 2006, p. 187). It is in this sense that I use the idea of “ethics” throughout this article, not as a cue for moral reasoning (although it may be a precursor) but instead as originating in our caring relationships with others.

### Method

The primary philosophical method of phenomenology is the reduction. Although the notion of the reduction has been articulated in a variety of ways, broadly it may be understood as a gesture consisting of two opposing moves: the epoché and reduction proper (Taminiaux, 2004). The epoché, or pause, refers to an ongoing attempt made by the researcher to set aside assumptions, theories, preconceptions, ideas and judgments held about the phenomenon under study, to gain access to the experience as lived. For this study, I constantly asked myself to suspend what I know and think I know about the experience of the nurse’s touch, ingrained during my own experience and learned through studying the texts of others. For example, I question if touch requires physical contact, and recognize that in my thinking about touch, I tend to emphasize healing and pleasurable touches versus hurtful or harmful touches. I recognize the need to intentionally check my research for the influence of this bias. The reduction proper follows the epoché, a positive movement toward the experience, to see its specific mode of appearing in the world (Taminiaux, 2004). Here, I consider what the moment of the nurse touching the baby might be like, as it is lived. Importantly, the phenomenological reduction is not reductionism, and it does not make the phenomenon smaller or lesser than it was; it is not removed from context. Rather it suggests to lead back to, from the French root *reducere* (Oxford English Dictionary, 2020c). As much as possible, we return back to the phenomenon as it gives itself to the world, before it was theorized, analyzed or explained.

Empirical phenomenological research relies on human science research methods, such as interview and observation, to explore an experience beyond that of the researcher (M. van Manen, 2014). I sought out nurse participants from four NICUs in Western Canada, using recruitment strategies of posters and emails. Interested participants contacted me directly and interviews were arranged at their convenience. In total, 10 currently practicing NICU nurses participated in the study. All were interviewed. The nurses shared experiences of nursing touch in the NICU during phenomenological interviews, oriented to gather pre-reflective experiential accounts of the nursing touches as they were lived. I paid particular attention to encouraging descriptions of specific moments, with as much detail as possible. Some participants were in their first few years of practice and others had practiced for decades. The nurses described varied experiences of touching babies and their families, providing a broad range of diverse examples of nurse-touch experiences. Interviews were audio recorded and transcribed verbatim.

In addition to interviews, one nurse submitted a written account of touch, and three nurses agreed to me observing their practice. For these observations, I joined each nurse at work on two occasions: each period of observation lasted between 3 and 4 hours, totaling approximately 18 hours. Close phenomenological observation can generate different kinds of experiential material, including that which is hard to articulate for the participant. While watching I became privy to small gestures that had not been referred to in interviews, such as stroking the baby’s cheek, gently bopping the nose, and light torso strokes while feeling the fontanelle. When I asked the nurses later about touches such as these, they often did not remember them. I wrote descriptive notes during and following the observation periods.

I worked with the gathered experiential material using thematic analysis. I read and re-read through anecdotes, interview transcripts and notes, wholistically and line-by-line attending to concrete descriptions of nursing touch. While reading, I identified lived experience descriptions of the nurse’s touch. Lived experience descriptions are descriptive moments that recollect the nurse’s experiences as pre-reflectively and concretely as possible, without personal opinions or generalizations about the experience (M. van Manen, 2014). Anecdotes were constructed by editing these descriptions in the direction of accessing the phenomenon by removing identifying and extraneous materials. From this material, I drew phenomenological themes. Phenomenological themes are not meant to summarize data, nor to generalize research outcomes, but to serve as heuristics meant to help uncover possible meanings that inhere in a particular moment of the nurse’s touch in the NICU. Lifeworld existential themes such as lived space, lived body, lived time, lived things, and lived relation were used to guide reflection on nurse experiences. I explored etymological and conceptual meanings to attend closely to the words and language used to describe the nurse’s touches. I have written and rewritten the text many times, aiming for resonance with the reader, in an attempt to convey some sense of the experience itself (M. van Manen, 2014). In deference to the infinite number of possible meanings associated with any human experience, phenomenological texts, including this one, should be read tentatively and with a questioning attitude (M. A. van Manen, 2017).

### Ethical Issues

Permission to conduct this study was received from the university health ethics review board and appropriate administrative and operational authorities. Consent was treated as an informed, ongoing, and evolving process. Before the interviews, the participant and I reviewed information about the study and any questions were answered before the consent form was signed. The interviews were held at a time and place chosen by the participant.

## Results

I remember one mom whose baby would cry and cry, and I would go into the isolette and contain the baby with my hands and tuck in the soother and rest my hand on her head. The mother said, “I feel like you have this magic touch, because every time I go in, she cries so hard. You go in, and she settles right away. What are you doing different than me?” I answer, “It’s really not me, it’s just what I’m doing.” Then I showed her how to try and sooth her baby with her touch.

A nurse may hardly be aware of the smooth movements of their own body while routinely involved in the NICU world, attending to a baby in daily care. Noticing their actions only if they are disrupted, for example, by dropping the soother or noticing a new rash on the baby. Until and unless they are gently uncovered by the observations of the mother’s parental gaze, nudging the nurse to attend to the ordinary moment afresh.

Parents who witness the skilled and soothing touches of a nurse likely do not believe the nurse is supernaturally conjuring comfort through their hands. To have a magic touch suggests that to someone else’s eyes, what appears to be a difficult task is handled with ease. For the nurse, responding to an unsettled baby’s cry is an ordinary and common part of daily practice in the NICU. The experienced nurse moves deftly: turning, re-positioning, containing, supporting, lifting, guiding the baby’s little body to find a comfortable position. Without needing to think it through, make a plan, or use an algorithm or other prescription, it seems the know-how to soothe a babe is expressed as coming from the nurse’s hands.

Often referred to as a phenomenological philosopher of the body, Merleau-Ponty (2012) asks if a routine movement (a habit) is “neither a form of knowledge nor an automatic reflex, then what is it? It is a question of a knowledge in our hands” (p. 145). Embedded in the muscles, tendons, skin, and flesh of the hands themselves, human touch seems to communicate in a vibrant and embodied complexity unrecoverable by words. It is difficult to express in spoken or written language what it is that touch does, or to describe how to touch the unsettled baby in a way that soothes and comforts, although some combination of words and demonstration might move someone closer to understanding. Without clearly spoken language, to an observer, these movements may look like magic, or at least effortless and natural. In this inquiry, however, we are continually asking what is the experience of touch like for the nurse? And we also recognize that this includes wondering what is the experience of touch like for the baby?

Even given a nurse’s tact and experience, there is no promise that any particular gesture or movement will “work” to settle the baby every time. For the nurse who returns to the baby to comfort and settle him over and over, the experience might be one of a practice, implying an ethical commitment. The nurse seems to act in response to an ethical appeal from the baby. As Langeveld (1983) reminds us, when we speak of encounters, it “does not mean we meet ‘others,’ but it means that we meet ‘each other’” (p. 6). There are ethics in these moments, welling up in the hands of the nurse, embodied in relation between nurse and babe.

The study participants described a manifold of examples of the varied touching gestures of an NICU nurse. These descriptions of touching moments have been organized around interpretive themes: the learning touch, the mark of touch, the missing touch, the gnostic touch, and the call of touch.

### The Learning Touch: Finding a Way to Hold the Baby

I am orienting to the unit and my preceptor says “just bottle that baby,” referring to this little premature baby in our assignment. I don’t know what to do, I have never even held a baby that small before, she seems so delicate. I say “I don’t know how to do that.” My preceptor walks over with me and says, “Well, just try picking her up.” I’m thinking, “How much do I need to support her neck? What do I do with the wires?” I put one hand under her head, pick her up and bring her against my chest with her head to my shoulder, my other hand under her bum, the cords that attach her to the monitor dangling.

We can appreciate how the learning nurse is focused on the many details of the task. The nurse may be self-conscious and unsure—I have never even held a baby that small before, taking in the physicality of the baby—she seems so delicate. Simply being in the NICU may make a baby seem more delicate and fragile, even when relatively well (M. A. van Manen, 2012). When first learning to retrieve a tiny premature baby from their bed, some preliminary, general knowledge is helpful; for example, the baby may have decreased tone and needs physical support, particularly under the neck as with all newborns. It is also important to know which cords or other attachments may be undone and which are critical. Ultimately, however, the way to learn how to pick up and hold a very small baby is by picking up and holding them. With practice, the skill becomes embodied and fades to the background for the nurse. Before, in between not knowing and knowing-how, is a rarefied interval of time and space where it is possible to catch a glimpse of what is happening for the nurse when lifting a tiny baby out of bed.

Openness, on the part of the nurse, seems necessary to allow for the possibility of moving toward and cradling such a small baby. The experience of holding the baby may seem constructed of separate, self-conscious gestures and physical maneuvering. The nurse may experience becoming newly aware of their own hands, where they are and how they move. We sense that the initial gesture is one of faith and requires courage—to reach for the baby and trust that they will figure out how to support, hold up, and bear the little one.

A movement is learned when the body has understood it . . . when it has incorporated it into its “world,” and to move one’s body is to aim at the things through it, or to allow one’s body to respond to their solicitation, which is exerted on the body without any representation. (Merleau-Ponty, 2012, p. 140)

Learning to hold a premature baby tenderly and competently is not only a matter of developing clinical skill but also cultivating an ethical receptivity to the comfort and needs of the baby. The gestures and movements of reaching toward and picking up the tiny premature baby inform and reform the nurse’s body.

From the thoughtful, careful attention to the task, we may discern that an experiential realization of the relational ethics involved is occuring. Ethics in this sense—a felt, embodied recognition that I (the nurse) cannot touch you (the baby) without being responsible for the touch, nor without being responsive to the baby (Manning, 2007). The experience of a novice nurse lifting a tiny baby up out of the plastic NICU cot for the first time reveals the ethical relation of the nurse and baby. In the nurse’s arms, the baby may seem less unknowable; the first embrace, though awkward, discloses to the nurse the possibility of moving closer to this baby in their particularity, as the baby’s nurse. The baby is wrapped up in the nurse’s arms and body, enmeshed and entangled in the nurse’s nursing world. Not simply fragile cargo, requiring careful handling, but a little human being fully implicated in the enfolding movement, weighing and pressing against the nurse, wiggling, moving, crying, sleeping, demanding the nurse’s attentive response.

### The Marking Touch: When Touch Lingers Long After Physical Contact

I scratch little Sam with my fingernail. Not just superficially. I draw blood. That’s how fast I am going. I am rushing through my assessment because I have so much to do. I feel horrible! I am instantly sweaty, and hot. I can feel the heat in my cheeks. I am so ashamed. I think to myself, “This is not OK. How could I let my nails get so long?” I hurt this child.

Unlike most touching moments experienced by the NICU nurse, an accidental scratch is unusual and may catch the nurse off guard. In a distracted rush, the nurse’s touch can injure the baby, blood signaling a wound in his tender, fragile skin. The appearance of bright red blood is experienced as an interruption, a rupture that jolts the nurse awake from the occupation of physical assessment, to see the baby as a child, as an other. In this moment of recognizing the injury, the baby may appear to the nurse differently than a second earlier, when he was the object of the assessment. The nurse encounters the wounded baby, perhaps in the same way she may encounter a hurt child she knows or any child who is not examined for their physiology. So delicate, the slip of a fingernail may break the skin and draw blood. To touch another always holds within it the possibility of harm. If an injury occurs, the nurse may viscerally resonate with the baby’s pain in a bodily response, flushed and shaken. Causing the unintended damage disrupts the nurse from a clinical attitude and awakens them to the being of the baby in such an immediate and compelling way that there is no option but to respond.

I was overcome with guilt for the rest of the night whenever I looked at him because of what I had done. I was so conscientious for the rest of the night, trying to be extra careful, feeling like my touch had to make up for hurting him.

The sensations of touch may linger after the physical contact is over—a painful touch that continues to hurt. These impressions of contact may mark not only on the baby but also the nurse. These may be fleshly vestiges felt in tired arms from holding and rocking a child to sleep, or a sore neck from bending over a lengthy dressing change. The lasting impressions of a touching encounter may also illuminate an experience of ethics, the touch that re-awakens the nurse to an ethical responsivity toward the baby. In these lingering reflections on an accidental, inadvertent touch, the nurse seems to experience a genuinely touching connection with the baby, one that persists after the physical contact is over. The nurse and baby remain connected, in touch while apart; the nurse now linked to the baby in attentive, apologetic, worrying, remorseful ways. Abruptly recognizing the baby as a child may be experienced by the nurse as being touched by the hurt baby, inviting the possibility of the nurse opening to sensations, perhaps of pain and anguish or sorrow. Although there is no way to heal the skin, or even explain and apologize, an ethical gesture is revealed in the impulse to make up for hurting the baby. We can imagine that the nurse may be forever marked, more attentive, more careful—yes, but also more aware of human frailty, of possibilities inherent in the intimate care of an other.

### The Missing Touch: Or Touching Without Physical Contact

Sometimes, a baby who is very ill needs to be pharmacologically paralyzed with a neuromuscular blocking agent to prevent them “fighting” the breathing machine.

Normally, if they’re awake and alert, I hold their hands and kind of play with them and make eye contact. With him, because he is paralyzed, I don’t. I resist running my hands through and combing his hair and even holding his hand because I don’t know what’s going to agitate him; he’s a very sick baby. Any time I do touch him I wonder if he’s tolerating my touch. When I reposition him, I wonder if my touch is bothering him. I hope the medications are keeping his mind quiet, but is he in there, upset?

Usually, the nurse draws near to the baby and reaches to touch and care for them. A wordless dialectic occurs as the baby feels the nurse’s touch and grimaces or wiggles or cries; the nurse adapts and modifies their touch in the lived immediacy of the moment, trying to find the most fitting of touching gestures, sensitive to the contingencies of the moment. For the experienced nurse, these bodily gestures tend to happen in a smooth, spontaneous, unremarkable exchange.

Conversely, the paralyzed baby cannot respond to any touch, laying still and unresponsive. Unable to touch the baby in the usual ways, or experience the baby’s physical response, makes the nurse feel somewhat uncertain about how to care for the baby. Even knowing that the baby is receiving sedatives and analgesics, the nurse worries that the baby is suffering when turned or shifted. Without grimaces, or squirms, or crying, or arching, the nurse does not know if they are giving care in a tactful way. Oxford English Dictionary (2020d) refers to both the sense of touch and a delicate sense of what is fitting in dealing with another. Mutual responsiveness helps to determine what is most fitting in any particular moment or situation. When the baby is unable to respond, the nurse may be left questioning what to do or how best to care for them.

In the absence of the baby’s corresponding gestures back toward the nurse, we might notice more clearly the formative part the baby plays in the nurse’s experience of touch, co-composing and co-constituting the physical connection between the two. Still, the potential of a touching moment may not be lost even when physical contact with the baby is compromised. Manning (2007) describes touch as a double genitive of two touches: “once in my gesture toward you and once in the experience of feeling your body, my skin against yours” (p. 11). The gesture that is initiated toward another with directionality is also touch. Rather than in spite of, but due to, not being able to touch the baby, the nurse becomes keenly sensitive to the experience of the baby, absorbed in wondering about the possible effects of limited touch when paralyzed and unable to respond. Refining physical contact to prevent potential discomfort to the child is an ethically conscious movement, attending to this individual child in the moment. Even without physical contact, the nurse may experience the connection to the ill baby as a touching one that begins and exists in a gesture of reaching toward the baby.

### The Gnostic Touch: The Possibility of Knowing an Other and Ourselves

As I lay my hand on his abdomen, his body shifts away as if in apprehension. I lightly lay my hand on his belly and wait a minute for it to soften, becoming used to the presence of the contact of my hand. As I feel him relax, I press gently with the pads of my fingers on one side and thumb on the other. I feel a soft firmness, like bread dough. I press a little more firmly and move gently and slowly back and forth, feeling for any masses, bowel loops. Then, I soften the pressure and move to feel for the margin of the liver with the side of my index finger on the right side; in the squishiness of the belly, the liver is firm, its inferior edge like a ridge pressing into my finger, just under the ribs.

The usual belly of a healthy newborn feels soft to palpation. The nurse’s comparatively large fingers sink into one side and then the other, alert to feeling any masses, lumps, or dilated bowel “loops” (regions of intestine filled with air or feces). In the contact of touch, the nurse may realize that their hands are too cold or too firm, or become alert to pathology beneath the skin. Palpating, assessing touches require a particular posture of hands, not digging in with fingertips, but pressing with the fingers’ pads to best feel for any unusual findings. Dense with nerve endings and with a capacity for fine dexterity, the human hands are well-suited to perceiving a wide variation of sensations. The touches that compose the physical assessment are necessarily tuned in, sensing and perceiving the baby’s body. To feel the texture of the abdominal organs and structures through the baby’s skin, the nurse may have to ease into the deep, searching touch of palpation, allowing time for the baby to relax and soften.

The Greek root gnostic means “pertaining to knowledge” (Oxford English Dictionary, 2020a). The nurse assesses the baby to come to know the baby better, both physiologically and by their reaction to the probing, pressing, feeling touches of assessment. The generalized knowledge required to perform a physical assessment is transformed through precise, perceptive touch to particular knowledge about one specific baby. Where does this baby press back, how do they move, does their liver sit a bit lower in the abdomen, is the brachial pulse steady, is their fontanelle tenser than yesterday? The nurse seems to be feeling beneath what the eyes may see, and tactilely exploring for what they expect to be present and for what they may find.

Nurse-philosopher Gadow (1985) reminds us that caring has a distinct moral position of “attending to the ‘objectness’ of persons without reducing them to the moral status of objects” (pp. 33–34). It is possible to objectify patients in health care, for example, when reducing a new baby born to their diagnoses: “the 28-weeker with hydrocephalus.” In the focus and concentration of assessing the baby, the nurse may experience the baby as an abdomen, a brachial artery, a skull. Perhaps it is less an experience of forgetting the examined body is a child and more that the child-ness of the baby fades into background.

During a period of observation, I watched as the eyes of a nurse met the eyes of the baby whose heart she was auscultating. The baby was small enough that when the nurse noticed the baby’s gaze, while still holding the stethoscope in place, she reached with her index finger to the baby’s nose and gave it a soft, playful tap. The nurse did not remember the experience when I asked about it later. The moment invites a wondering pause—noticing the baby’s gaze, the nurse effortlessly switches from auscultating the baby’s heart sounds to another touching moment. It is as if the baby’s gaze touches the nurse who responds with a tender, playful touch. Perhaps any distinctions between the gnostic touch and the more relational touch are mellowed and softened in the nurse’s experience of them, not necessarily changing from one to the other, but merging and blending. As the nurse assesses the baby by touching, feeling, listening, and observing, they perceive corresponding sensations of pressing, grazing, squeezing, tickling in return. The nurse does not need to think about or give cognitive consideration to these touches to experience being in contact with the baby; rather, they are felt. Coming to know another through the touches of assessment may be experienced as embodied intersubjectivity.

### The Call of Touch: Drawn to Hold

A nurse recalls a baby arriving with the transport team. The baby had a significant heart defect and was profoundly unstable and unwell.

His parents are still en route from a remote community. His blood pressure just keeps tanking, just dropping. We push fluid, transfuse blood, infuse epinephrine boluses, only to be followed by more medications. There is a lot of intense activity . . . but nothing is helping. We arrive at consensus that nothing is going to work, the resuscitation ends. As the rest of the team left, my first instinct is to pick him up and I hold him close in my arms. I don’t think about whether or not I should, just that this baby is dying alone.

The moments of trying to stabilize and resuscitate a critically ill newborn can be congested, hectic, and demanding. Health care practitioners, intravenous poles, and equipment crowded around the small bed, trying to save the baby’s life. These urgent touches require their own expertise; they are not the gentle or tender or comforting touches that we might expect in newborn care. These touches, the touches of revival and survival—pressing and compressing the chest, straightening and stretching limbs, poking and pulling skin—happen at a pace of disciplined efficiency, constantly calibrating with physiological feedback, how’s the blood pressure? What’s the oxygen saturation? In such moments of striving to save the life of the baby, the team of health care practitioners do not seem to reflect on whether or not they should touch this child. Instead, these touches are given in response to the demand of the moment—saving the small baby.

As the end of the code is realized, the practitioners slowly draw away from the baby. Chest compressions cease, stethoscopes hung up, intravenous pumps stopped, still full syringes of medications left on the counter, ungiven. The float nurse who had been helping goes to assist others; the physician, respiratory therapist, nurse practitioner all similarly leave to assess another child or to speak to families, or to write notes, or other work. Without parents, eventually, the nurse is left with the baby at the now quiet bedside, the urgency seemingly over. And yet, a different kind of ethical necessity occurs. In that moment, the nurse realizes the child is dying alone and is struck by their alone-ness, with no parent present to hold them. It is as if the nurse recognizes the child needs to know the presence of another and is spontaneously compellingly drawn to hold the child. To hold, from the Old English healdan, is to keep watch over (Oxford English Dictionary, 2020b). The NICU nurse is always already “watching over” the baby in their care. But when parents are not present, they may also stand in a relation of in loco parentis to the baby, their nurse-responsibility augmented by the temporary responsibility of filling in the place of a parent, assuming for a moment the sense of caring for this child as one’s own. The baby dying alone utters a call of need and the nurse experiences being claimed by this child who is dying, called to foster and watch over him, revealing the close-up, temporal, situational, and spontaneous nature of ethics embodied.

## Discussion

It may seem odd to have embarked on an investigation of the NICU nurse’s touch and the relational ethics wherein, without any mention of the right or wrong touch. In a Levinasian sense, the purpose of this research was to attempt to make contact with the ethics of touch even before we consider what a morally correct touch might be in a particular situation or context. It is not that a normative quality cannot be observed in the phenomenological examples shared here. Rather, this is an attempt to resist the development of a health ethics that exists primarily at a cognitive level, which sets the body aside, flattening the significance of our daily lives into representations (Fielding, 1998). An embodied ethics is a vibrant, corporeal, felt ethics, perhaps a result of the body’s amazing ability to adapt to our world, not merely through our rational abilities, but through our senses (Merleau-Ponty, 2012). Touch is the nurse’s primary connection to the textures, temperatures, and topographies of the baby and the NICU world (adapted from Montagu, 1986).

Exploring the touching gestures of NICU nurses discloses the relational ethics at the heart of caring practices. Surely, these gestures vary in purpose and character, each with its own particular pace and rhythm: the charged, acutely focused, algorithmically guided movements of resuscitation; the slow, awkward, self-conscious gesture when learning to pick up and hold a premature baby; the methodical, systematic techniques of physical assessment; the smooth, spontaneous swoop of gathering the dying baby into arms; even the rushed, distracted, accidental scratch. Yet fundamental to all is an embodied ethics expressed in the mutuality of touch. To consider how touch brings us close to one another; we might read Merleau-Ponty as Stephen David Ross (1998) does, by “recognizing bodies, touch, and proximity as the places where beings open to each other, interrupting the solidity of everyday life” (p. 9).

When interrupting the solidity of life through touch, we become aware of being in constant relation with an other. The nurse is not only the one who touches the baby but also the one being touched by the baby, in the inherently ethical experience of touch—making relational contact with another. Sally Gadow (1984) reminds us that “both sides [patient and nurse], as it turns out, have something of value to give the other—a fact overlooked” (p. 69). Perhaps in the context of the NICU, what the baby offers the nurse is physical vulnerability that demands a response. The baby cannot verbally proclaim trust of the nurse, cannot convince the nurse to care for them, squeeze their hand in return, or make a bargain, or justify, or advocate. They can only make an embodied claim, by their very existence.

The NICU nurse being claimed by a child may be likened to experiencing a spark of ethics, a felt impulse reaching toward the baby. Whether this impulse is to pick the dying baby up, or another gesture of touch, the nurse is not acknowledging ethical codes, nor ascribing to philosophical theories of ethics; instead, they are open to the baby’s appeal. Conceiving an embodied ethics implies that we are not bound only to rules and duties but that an evolution of ethics is “the ongoing transformation of expressive bodies toward spontaneous right action” (Mazis, 2006, p. 188). Nurses have long recognized that touch is more than skin-to-skin contact, involving a “multi-dimensional gestalt” along with voice, posture, and affect (Estabrooks & Morse, 1992). Sometimes touching transcends physical contact and the nurse can both touch and be touched without physical contact (as with the paralyzed baby) or with “broken” touch (the scratch).

Living through the physical distancing required during the pandemic has uncovered a tension involved with being physically close. Many of us have discovered the possibility for meaningful encounters without touching, by keeping distances and mediated online, while recognizing that nothing really takes the place of close, touching gestures in our relationships and lives with others. A question that remains may be whether or not there can be physical contact, the most basic understanding of the definition of touch, without a genuine touching encounter with an other. In particular, is the accidental scratch a nursing touch at all, as it lacks the characteristic care and attention we expect of a nurse?

By including the scratching touch as an example of the way nurse’s experience touch, my purpose was to illuminate the genuine closeness between the NICU nurse and the baby; both are vulnerable to the risk that human touch poses and to its lingering influence potentially extending the sensation of touch beyond and after physical contact. “Sensations are not governed easily; they reach deeply into and around the body, creating space and altering the trajectories we thought we could delineate cleanly, legitimately, between sensing bodies in movement” (Manning, 2007, pp. 66–67). Bodily skills and practices of nurses do not occur fully formed; nurses cannot hope to never harm a baby with their touch. Expertise in practical skills develops over time and through practice. I have attempted to show the inherent ethics of the nurse’s touch and emphasize that it is more than a physical tool of task performance. Dreyfus et al. (2009) explore a phenomenology of expertise for ethical comportment in nurses in part through an analysis of Carol Gilligan’s moral maturity scholarship. They suggest “the highest form of ethical comportment consists in being able to stay involved and to refine one’s intuitions” (p. 328). By staying present, the sensations of harming the baby wrapped around and drew the sympathetic nurse back to the baby, close in a (nurse-) touching response of ethical attendance.

## Concluding Thoughts

In the nursing literature, as well as in practice and in society more generally, there is not enough attention to the risks of the lack or absence of touch. Too often, our collective response is to forbid touch—to restrict and condemn all touches—to prevent harmful touching. Poet David Whyte (2018) reminds us that

to forge an untouchable, invulnerable identity is actually a sign of retreat from this world; of weakness, a sign of fear rather than strength and betrays a strange misunderstanding of an abiding foundational and necessary reality: that untouched, we disappear. (p. 223)

The corollary of this, of course, is that touched, we appear.

For the NICU nurse, might we think of the ethics of touch as one of appearing? Through touching gestures, the baby is revealed and appears to the nurse in their full subjectivity. We can only thoughtfully speculate about what the baby is experiencing, but the touching encounter strikes the nurse as a reminder to constantly be concerned for the way the baby might experience the nurse’s appearance to them.

Attending to the experience of nurses’ touch gives us more clues to understanding the experience of an embodied relational ethics implicit to the practice of nursing and other health care practitioners. The effects of touch may go undocumented and unrecognized in many nursing situations. By attempting to articulate these practices, the hope is that the significance and contribution of touch and the embodied wisdom of the nurse might be recognized and brought forward to our individual and professional consciousness, conversations, and curricula.
